# Did we create brave spaces? A realist evaluation report on simulation-based faculty development workshop in equity, diversity, inclusivity, and Indigenous reconciliation

**DOI:** 10.1186/s41077-025-00346-2

**Published:** 2025-04-05

**Authors:** X. Catherine Tong, Sonaina Chopra, Hannah Jordan, Matthew Sibbald, Aaron Geekie-Sousa, Sandra Monteiro

**Affiliations:** 1 Department of Family Medicine, McMaster University, Kitchener, ON Canada; 2 McMaster Health Education Research, Innovation & Theory (MERIT) Centre, Hamilton, ON Canada; 3 Simulated Patient Program, Waterloo Regional Campus, McMaster University, Kitchener, ON Canada; 4 Department of Medicine, McMaster University, Hamilton, ON Canada; 5 Midwifery Graduate Program, McMaster University, Hamilton, ON Canada

**Keywords:** EDI, Indigenous reconciliation, Simulation-based education, Faculty development

## Abstract

**Background:**

Creating Brave Spaces (CBS) workshops are designed by an interprofessional team of health professions educators to empower faculty members to disrupt microaggressions in the clinical teaching environment using simulation-based education design, where actors were trained to portray sources of microaggressions.

**Methods:**

The CBS team delivered eleven workshops addressing five categories of biases in various contexts during 2020–2024 engaging hundreds of participants. The team recruited participants to conduct semi-structured interviews. Records from team meetings and facilitator focus groups were collected and reviewed. The dataset was subjected to thematic analysis focusing on the participants’ experience in the workshop. Themes were presented in Context-Mechanism-Outcome statements informed by the realist evaluation framework. Subsequently, the results were verified with participants.

**Results:**

Nine participants volunteered to be interviewed 2 to 12 weeks after attending the workshop. The interview scripts, totaling about 60,000 words, provided a rich picture of faculty members’ backgrounds and experiences. Thematic analysis yielded the following results. Simulation-based education design empowered faculty members to overcome barriers and progress in their skills. During the immersive experience, participants benefited from a rare opportunity to practice aligning their values with their actions. Those who experienced microaggressions as victims or passive bystanders in their past experienced heightened emotions. Faculty members agreed that disrupting microaggressions is an important part of their work. They navigated the tension between “calling in” the source of the microaggression, being mindful of power dynamics in the simulated cases, and “calling out” the harm of microaggressions by holding the source accountable. Some recounted successes in managing subsequent incidences of microaggressions in their clinical teaching environment. The results were validated by a member-checking process, and further supported by recorded conversations during team meetings and facilitator focus groups.

**Conclusions:**

Health sciences institutions’ stated strategic goals in inclusive excellence, although widely accepted by faculty members, are challenging to operationalize in the moment of a microaggression. Participants practiced this skill using simulation-based education design and reported significant and positive impacts.

## Background

Since the tragic deaths of George Floyd in the US and Joyce Echaquan in Canada, an Equity, Diversity, Inclusivity, and Indigenous Reconciliation (EDI-IR) education reform has gained momentum in health professions education [1–5]. Many institutions name and center EDI-IR as high-level strategic objectives. In Canada, we open formal meetings with land acknowledgements. We create a curriculum for learners to address EDI-IR learning objectives. We strive to apply an equity lens in all aspects of organizational life including admissions, recruitment, and promotion. Recognizing faculty members as an important part of this reform, a competency framework for medical teachers has been established [6]. However, individual faculty members rarely have opportunities to act on these values. Simulation-based education can be leveraged for these learning objectives [7–9], while few focus on training faculty members to manage microaggressions as they arise in the clinical teaching environment [10, 11].

As originally coined by Chester Pierce and later defined by Derald Wing Sue, “racial microaggressions are brief and commonplace daily verbal, behavioral, or environmental indignities, whether intentional or unintentional, that communicate hostile, derogatory, or negative racial slights and insults toward people of color” [12, 13]. Many marginalized groups, which include racialized people, experience these subtle and casual put-downs and indignities. Ackerman-Barger et al. outlined that in any given incidence of microaggression, there are three perspectives: the source, the recipient, and the bystander(s) [14]. Anyone in the clinical teaching environment, including faculty, staff, learners, patients, and family members, could find themselves as the source, or recipient, or bystander. In social science, bystanders have been recognized to have a significant role in combating various forms of injustice; bystanders who actively intervene are called “upstanders” [15]. The series of cognitive and behavioral steps upstanders must undergo to take effective action has been studied [16]. Training and practicing these steps can therefore reduce the burden and increase the chance of responding effectively in real-life incidences.

In 2020, we launched the Creating Brave Spaces (CBS) workshop series at McMaster University to empower faculty members to disarm microaggressions as upstanders in the everyday clinical teaching environment. We recruited actors as sources of microaggression, playing the roles of senior colleagues, peers, or patients. We placed participants in simulation to respond to the actors. We reported on our early experience of delivering this workshop separately [17]. In this paper, we report on the results of a program evaluation study on the CBS workshop series.

## Methods

### Qualitative approach and research paradigm

In this study, we adopt a constructivist orientation. We believe that each participant experiences the workshop differently based on their positionality, current environment, and interactions specific to each workshop. To analyze the results, we apply reflexive thematic analysis to our data [18]. We then organize the findings through the realist evaluation framework [19].

Sociologists Ray Pawson and Nick Tilley developed the realist evaluation framework to explore the underlying processes by which programs may achieve their outcomes, beyond the binary question of whether an intervention “works” [19]. It asks: “What kinds of educational interventions will tend to work, for what kinds of learners, in what kinds of contexts, to what degree, and what explains such patterns?” [20] To develop answers to these questions, a realist evaluation study asks learners to describe their context, which could include their work environment and the training location. The study also creates data to describe various mechanisms that led to the intended and unintended learning outcomes. These data are formulated as answers to the questions above to create “program theories” in the context-mechanism-outcome (CMO) format [20]. In faculty development, faculty members come from various contexts and may access different mechanisms to achieve a variety of outcomes. For example, a CMO statement in the context of our study might be: faculty members who are motivated to support EDI-IR objectives (Context) can engage with simulation-based training (Mechanism) and become more confident in managing microaggressions (Outcome).

### Researcher characteristics and reflexivity

#### Reflexivity statement

We are an interprofessional group of health professions educators at McMaster University in Canada. Some of us are faculty members (SM, MS, XCT) and others are staff members (HJ, SC, AG-S). XCT is a family physician, emergency physician, and clinician educator with a focus on faculty development. MS is a cardiologist and clinician educator with a focus on simulation-based education and undergraduate medical education. SM is a PhD educator and researcher with expertise in assessment strategies and simulation-based education. HJ and AG-S engage with simulation-based education as part of their portfolio. SC is a research support staff member. The majority of our team share a training background in biomedical sciences, which is associated with a positivist epistemology. However, for this study, we adopt a constructivist epistemology, which is consistent with the qualitative research methods we employed. In addition to a diversity of education experience and expertise, we are a diverse group in racial, ethnic, gender identities and sexual orientation. Each of us brings our professional network, the perspectives of our personal communities, and our own lived experiences. For example, simulation cases reflected both careful design and elements of real-life incidents. Debrief conversations represented both literature on the topic and perspectives from a marginalized identity. Data collection, analysis, and interpretation were informed by our professional and personal positionality.

XCT was supported by the MERIT Faculty Fellowship in 2022-2023 to launch the project and volunteered her time after 2023. SM and MS volunteered their time throughout the project. HJ, SC, and AG-S either received permission to work on the project during work hours or volunteered their time outside of work hours. Although education and research are central to each of our roles within the institution, none of us holds a portfolio that relates specifically to EDI-IR. The team applied for grant funding whenever possible to sustain the operational cost of the workshops.

As a team, we find alignment of our personal values with the institution’s objective of inclusive excellence, and we see this grassroots project as a way to operationalize inclusive excellence in the everyday clinical teaching environment. While we leverage the momentum of EDI-IR education reform that has been built in academia in recent years, we are concerned about the emerging backlash and more recent complete reversal in EDI progress [21, 22]. We hope to put forward evidence of the impact of this workshop series to stimulate ongoing conversations related to EDI-IR, and we need continued institutional support. Ultimately, we aim to make progress in our shared goal of a vibrant and safe workplace in our clinical teaching environment.

### Ethics

The study proposal was reviewed by the Hamilton Integrated Research Ethics Board (HiREB-15339) in September 2022. It falls within quality improvement/program evaluation, and as such was granted a waiver from a full review.

### Data collection methods and instruments

#### Workshops and case construction

In constructing cases, we adapted the format to fit within the constraints of the learning context. For example, the way in which participants engaged with a simulated case differed when the setting was a virtual conference compared to an in-person faculty development all-day retreat. Because faculty members are not routinely engaging with this material, we allocate 30 to 45 min to define microaggressions and suggest one approach to interrupt microaggressions, i.e., ARISE [14]. We also include basic background information related to the specific microaggression for each workshop. We then present the simulation component of the workshop, which would be at least 30 min (1 case) and up to 90 min (2 cases). Cases are designed to fit the reality of the session. For example, a virtual curricular meeting would fit a virtual workshop. The case scenario and actor’s script are based on real-life incidents reported by members of the marginalized community. After initial scripts are drafted, cases are then vetted by team members and may undergo further revision. For example, a transphobia case was written based on real events by a trans-woman and staff member. The anti-indigenous racism cases were written by an indigenous faculty member. Cases are then edited to fit the format of the workshop. In all cases, participants are in a group interaction with a trained actor in a clinical teaching or administrative environment. Participation is voluntary. The actor plays a senior colleague, a peer, or a patient. They are trained to make casual comments that are microaggressions towards a marginalized group. The comments are written in such a way that it is ambiguous whether there are victims present in the scenario, eliciting a bystander response amongst the participants. The actor is prepared to be interrupted by the participants and may choose from a collection of additional arguments that further challenge the participants. The scenario may pause, rewind, or move forward based on the needs of the participants.

#### Level of difficulty

The intended audience of this workshop is clinician teachers and education program staff members who espouse EDI-IR values but not teachers or content experts in this subject. The cases are designed so that the microaggressions are clearly identifiable, following the presentation related to definitions. The skill required of the participants is to interrupt the source of the microaggression by any approach they deem appropriate, whether they choose to use the presented approach or not. After presenting the first workshop, we further lowered the level of difficulty by printing the ARISE approach and distributing it amongst the participants as cues, so that there is minimal cognitive barrier in performing the skill.

#### Topics

Because we had intentionally set out to address a collection of major topics, we produced the first two cases based on the availability of the writers who are transgender and indigenous, respectively. Subsequently, we recruited writers on other major topics in EDI, including racism, gender bias, xenophobia, and ableism. We expect to continue to grow our library of cases to meet the needs of various groups in our community.

### Development of interview guide

Using the realist evaluation framework, the team developed a semi-structured interview guide, designed to prompt participants to share their positionality, describe their professional context, including their knowledge of local EDI initiatives, recall their experience in the workshop, and reflect on their learning outcomes. Following the CMO format, we first asked participants about their work environment and their personal engagement with EDI values. We then asked them to recall the events and emotions during the simulation, their reaction to the cases, what they were thinking about while participating, and how other participants responded. And finally, we probe them on immediate and longer-term outcomes from the workshop, including changes in their attitude or responses to similar incidents.

#### Pilot interviews and interview guide iteration

Two workshop participants were recruited to pilot the first draft of the interview guide with a trained research assistant (SC). The transcripts were produced by a professional transcription service with a confidentiality agreement with the study team which was able to produce anonymized transcripts. SM and XCT reviewed the transcripts against the original recording to ensure accuracy. Occasionally, the transcription service missed words; we were able to fill them in after listening to the recording. The team refined the interview guide to gain greater insight into the mechanism based on the review of pilot interviews. For example, we added specific questions about whether the experience felt “real”, to what degree did participants experience “risk” in deciding whether to speak up, as well as whether the participants felt “triggered” during the simulation.

#### Additional participants’ interviews, focus group meetings

We proceeded to recruit additional participants for interviews as we delivered workshops. Volunteers were verbally invited to participate at the end of a workshop. Participants were recruited from three consecutive workshops at the Waterloo Regional Campus Faculty Retreat (October 2022), Niagara Regional Campus Faculty Retreat (February 2023), and Hamilton post-graduate medical education family medicine training site Lunch and Learn (March 2023) event respectively. These were the first three workshops delivered to McMaster faculty members. In total, 9 faculty members participated in the study, a sample drawn from 43 total participants at these three workshops. The cases presented during these workshops were Gender Neutral Pronouns and Orange Shirt Day 1. These cases address transphobia and anti-indigenous racism, respectively. A complete catalog of cases offered at the CBS workshop series at this time, with case synopsis, is shown in Table 1.

**Table 1:** CBS workshop case synopsis

Topic	Writing and review process	Case synopsis
Transphobia	The ideas came from university staff members and leads in EDI portfolios with lived experience. Each idea was based on real-life incidents. The ideas were adapted into cases by the CBS team, reviewed, and approved by the faculty members.	“**Gender Neutral Pronouns**”: in a curricular meeting, a senior faculty member rants about the workload related to changing all pronouns in the teaching cases catalogue to the gender-neutral “they/them.” “**Where is My Pride Month**”: in a team meeting, as Pride Month approaches, a senior physician laments about the focus on transgender rights in the media. “**Trans Heath Course**”: in a staff meeting, a senior nurse disagrees that transgender health is an important topic in nursing continuing education.
Anti-indigenous racism	The ideas were submitted by an Indigenous faculty member who leads an Indigenous health portfolio at the undergraduate MD program based on her personal experience. The ideas were adapted into cases by the CBS team, and reviewed and approved by the faculty member.	“**Orange Shirt Day 1**”: In a curricular meeting, a senior leader rants about having to attend an Orange Shirt Day ceremony in place of other planned commitments that they perceive to be more important. “**Orange Shirt Day 2**”: In a 3-part series, an administrative staff misses the point of Orange Shirt Day by ordering orange coloured shirts from a non-indigenous business. The Indigenous community representative confronts the planning committee.
Anti-Black racism	The ideas were submitted by black academic faculty members who are published experts in anti-black racism. The ideas were adapted into cases by the CBS team, reviewed, and approved by the faculty members.	“**Where Is He**” in the OR, a senior nurse criticizes the social justice activism of a black trainee while waiting for him to arrive “**The New Student**”: in a community-based teaching clinic, the manager uses stereotypes to describe a black student who is joining the clinic the following week
Sexual harassment	The idea was submitted by a faculty member who witnessed this incident in clinical practice.	“**Follow-Up Appointment**”: in an outpatient teaching clinic, a patient sexually harasses a resident during a medical appointment
Xenophobia	The idea was submitted by a faculty member who witnessed this incident in clinical practice.	“**The IMG**”: in an outpatient teaching clinic, a patient is hostile towards an internationally trained resident and refuses to discuss her medical concerns

Following participant interviews, we organized focus group meetings where the conversations were recorded and transcribed. Study team members, guest facilitators, and actors met to reflect on their experiences with the workshop, where the conversation followed an open-ended, semi-structured Q + A format. These transcripts were included in data analysis, as well as all study team meetings.

### Data analysis

#### Coding and thematic analysis

All transcripts were produced by the same professional transcription service and stored on an institutional licensed shared drive with 2-step authentication. We conducted data analysis using the process outlined by Braun and Clarke [18]. We started with open coding, where notes were placed as comments attached to participants’ statements. Team members commented freely and identified interesting or important quotes, which were discussed at team meetings. Subsequently, two team members (XCT, SM) reviewed each transcript iteratively, reading and re-reading, to conduct line-by-line coding in a systematic fashion across the data set, taking care to discuss and reflect on potential data items. We used Post-it notes (© 3 M 2024) to facilitate visual organization of the codes and placed them spatially to consider relationships amongst them.

All codes were then entered into an Excel (© Microsoft 2024) document and linked to extracts in the form of quotes. The codes that centered around the same ideas were then collated into potential themes. The themes were reviewed and appraised with another review of the entire dataset. While verifying the themes, additional codes that were missed in the first reviews were added to the codebook. Preliminary analysis results were presented by SM and XCT to the rest of the team for feedback.

Transcripts from team meetings and focus group meetings were reviewed after participant interview transcripts. These documents were not specifically subjected to thematic analysis, but we were able to validate and accentuate themes already identified by participant interview transcripts. For example, focus group interview transcripts corroborated events described by participants. Actors shared their observations, echoing participants’ responses. Guest facilitators described difficult conversations during the debrief and shared their perspectives on the strengths and limitations of the education design.

The final step of data analysis involved constructing the program theories in the C-M–O format. Based on the themes generated in this process, XCT and SM assigned elements of context, mechanism, and outcome. The team reviewed and revised these program theories, resulting in five final C-M–O statements.

#### Member checking

As a last step in enhancing the trustworthiness of the results, we shared the results in a narrated online video format with the study participants, 12 months after the interviews had concluded. The video is a recorded PowerPoint slideshow that reviewed the background and the main findings of the study. We solicited their feedback using an online survey tool and collected the responses to be included in the data set. In this survey, we asked participants whether the results accurately reflected their experience. We asked them to critique how the results resonate or differ from their own recollection. We provided another opportunity to share if the activity made an impact on their practice.

## Results

We present the results in the following fashion. We describe the dataset, followed by five themes informed by the data. For each theme, we propose a C-M–O statement in the style of realist evaluation, paired with quotes that support the statement. A summary of the themes is presented in Table 2. We offer implications for health professions education in the “ Discussion” section.

**Table 2:** A summary of CMO statements

Context	Mechanism	Outcome
Resources provided during the workshop	Given opportunity to practice in simulation	Participants still found it difficult to perform the skills
Motivation to operationalize their values	Given opportunity to align values with concrete action	Experienced relief and excitement
Lived experience of victimhood and being a passive bystander	Given opportunity to manage the interaction as an upstander	Experienced empowerment
High productivity pressure	Simulated experience of encountering microaggressions while under productivity pressure	Anticipate the task to manage microaggressions while managing productivity pressure
Interpersonal dynamics and valued relationships	Simulated experience of encountering microaggressions from a source with power	Gained experience in navigating the tension of ensuring accountability and maintaining important relationships

### Dataset

Nine participants were interviewed 2–12 weeks after attending the workshop. The interviews resulted in a transcript of 60,000 words. We also reviewed the transcripts of four team meetings and two focus groups. Finally, we collected 8 responses from the member-checking survey. The responses from the member checking survey strongly agreed with the synthesis of our team.

**Fig. 1:**
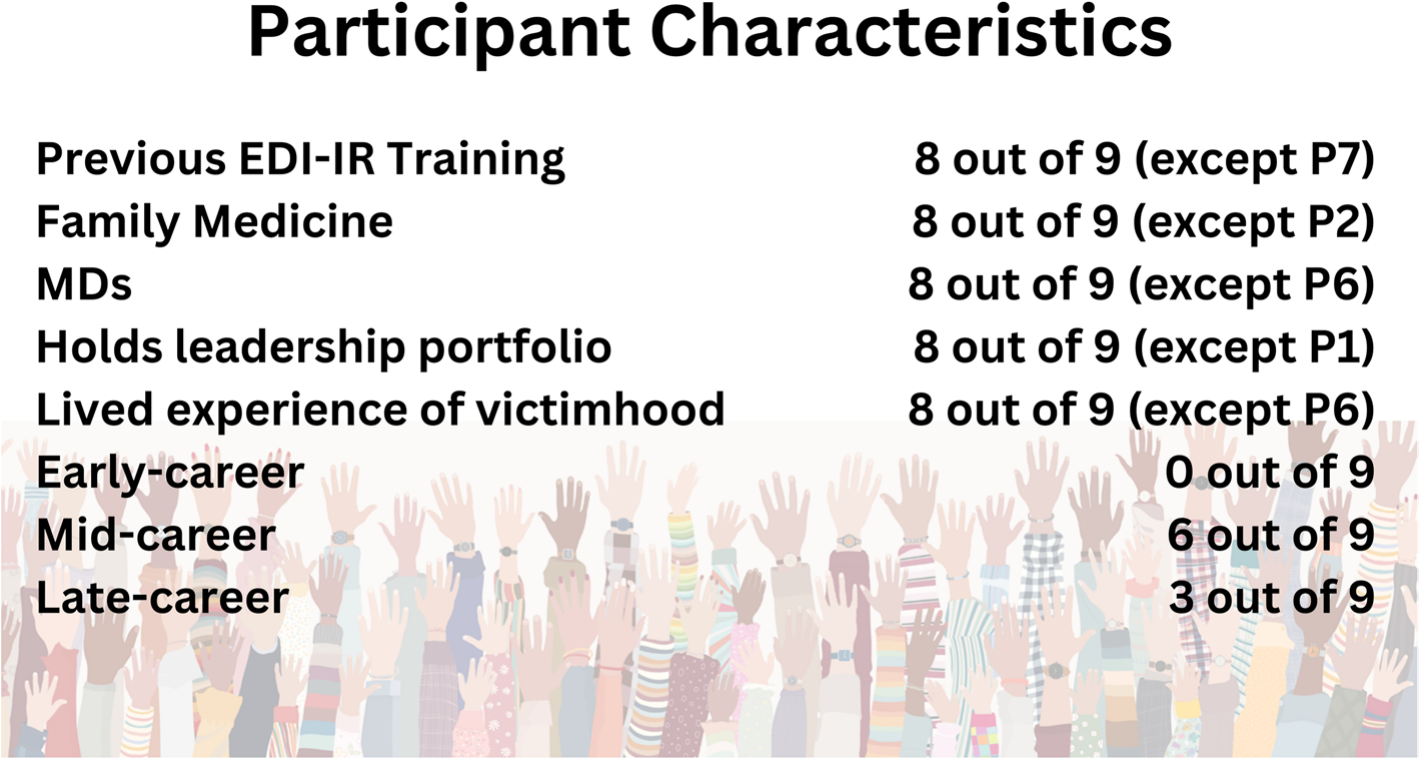
Study participant characteristics

For a summary of participant demographic data, please see Fig. 1.

### Synthesis and interpretation

**Program theory 1**: provided with resources and the opportunity to deliberately practice, faculty members generally still found it difficult to perform the skills in interrupting and disarming microaggressions.

The steps involved in interrupting a microaggression are complex and difficult to execute [16]. Most of the time, participants quickly identified the microaggression. However, to interrupt a microaggression proved to be more challenging. Participants often appeared frozen as they processed and considered their options, even after having been provided with a stepwise algorithm to do so. When facilitators deployed “time-outs,” participants had a chance to regroup and debrief. Facilitators ask questions like “what is going through your mind”, “how do you feel at this moment”, and “what would you like to say to your colleague?” Participants used these cues and the break to organize a response. As the scenario was acted out repeatedly, participants had the opportunity to try multiple times, either by “rewinding” the case or through a different case. Over time, they performed with more conviction and less hesitancy, indicated by tone, collaboration between participants, and shorter times to respond to the aggressor.

Example quotes:

“And I think when we first, when it first started going, it's like you know somebody is saying something that you don't agree with. And you think is either you know is racist or what have you. But then everyone stays silent. And you can feel it and you kind of feel that energy of where people want to say something but they don't know what to say.”(P1).

“But I really think you have to be in that scenario to know how you will react.” (P3).

“I remember using it to the best effect in Niagara… literally going okay pause, what are we thinking? And then you actually give the people the opportunity to go okay what was this ARISE…Every time they need that pause it seems.”(team meeting transcript, April 2023).

“…my observation of that has been that it is not often our learners who struggle with that because they are getting some of that in the curriculum now, right, as you well know. It is our faculty who have been around for a long time. And I don't mean that in a stereotype of it is always the older white guy. It's not. But I think that they struggle even with just, and I have heard this from many people in the work that I have done at … as well is that they struggle with the I don't know the language. And I don't know what to say and I don't want to get it wrong. And you know there is a lot of that which they feel that they haven't sort of grown up with.” (focus group transcript, July 2023).

**Program theory 2**: through simulation, faculty members experienced relief and excitement at the rare opportunity to align their stated values with concrete action towards equity and inclusivity.

Participants noted that this workshop had been a rare opportunity to practice supporting people who are targeted by microaggressions, which allowed them to act on their own values. They noted that, after having successfully practiced the skill, their emotions during simulation changed from a negative state to a positive state.

Example quotes:

“And one thing that I took from the workshop as well is like you have to honour your own values. And when you are experiencing these things. And not saying something you are not honouring your own values.” (P5).

“I think sometimes the not saying anything sticks at me longer than the saying something. At least if you say something you can go back and be like ‘oh I could have said it differently’ or ‘I wish I maybe would have said it like this’… and then you can self-reflect.. (P6).

“And I actually took the moment and said I want you to watch what I am going to do here as staff. You can't do this because you are a resident. And they are not your patients. So, you don't have the same power in this situation that I have. But check out what I am going to do. And I did it in front of her.” (P8 recounts how she managed a situation where a resident was victimized by microaggressions from a patient, in her practice, after attending the workshop).

**Program theory 3**: faculty members who have experiences as the victim or as a passive bystander of microaggressions managed heightened emotions when attempting to interrupt microaggressions.

Participants described strong emotions during the workshop simulation experience. The words used to describe their experiences included: “intense”, “uncomfortable”, “anger”, “frustrated”, “shocked” and “anxious”. Eight out of nine participants described in detail their personal experiences as victims of microaggressions through various marginalized identities (gender, sexual orientation, race, and ethnicity). We also gathered stories of persisting guilt related to witnessing microaggressions passively as bystanders. We hypothesize that these experiences may have been unprocessed and unresolved, and recalled during the simulation. This recall may have led to strong reactions to the scenarios and may have affected their performance. It may have contributed to the initial flight/freeze response. Their reaction frequently turned to “fight”, however, when they recognized that they have multiple opportunities to act during simulation. In doing so, they stepped out of their prior narrative of being a victim or a passive bystander and moved towards effective action. We believe the workshop experience helped free the participants from their prior negative experiences.

Example quote:

“Yeah, because you see micro-aggression and being a person who lived these situations there is some intense emotion that can build up in your brain or inside. when you're in the workshop you reflect, you are thinking that there are lots of thoughts that come to your mind. Lots of experiences flashback. Yes.” (P4).

“And you definitely feel guilty. There are definitely scenarios where I should have said something. Like how come I didn't say something… And that was kind of interesting because I have never really done that before where you kind of just say well why can't you do it again? Like why does that have to be the end.” (P1).

**Program theory 4**: faculty members accepted that addressing microaggressions is an important aspect of their work despite considerable productivity pressure to complete other important tasks.

Faculty members of medical programs are under significant productivity pressures in both clinical work and teaching responsibilities. The need to “get through” tasks and workdays is paramount and palpable even during a simulated activity. However, when asked directly, most participants affirm strongly that managing unexpected microaggressions is an important task and part of their role as teachers and leaders.

Example quotes:

“…the discomfort when things were not going to plan… a pressure to bring the meeting back on track—is this productivity pressure? or generally a fear of chaos.” (P7).

“So, we could theoretically be hijacking another agenda with this. How would you contain it kind of thing. if we are in our place as leaders, and something like this was said and no one, no one responded. And we see, you know, there is plenty of stuff to do in the next hour kind of thing, what is, I say as the chair, how do we recalibrate ourselves around an occurrence like this?” (P9).

“…it is something that I think is very relevant to day-to-day practice in family medicine. And it is definitely something that, um, it is helpful for me to help guide my learners too as they are approaching situations where you know EDI becomes like an active part of the conversation.” (P5).

“I feel like it is an unnecessary thing that we need to deal with. And it can affect the doctor-patient relationship. It can affect the educator learner experience or relationship. So, I just find it unfortunate when those circumstances come up. So I feel it is uncomfortable because it just basically taints the experience. And having to address it just makes that uncomfortable to have to deal with.” (P2).

**Program theory 5**: when faculty members challenge the source of the microaggression, they feel comfortable starting with a compassionate and curious stance, while recognizing the importance of protecting the victim and ensuring accountability.

In the face of harm, faculty members recognized the importance of accountability and interrupting harm to potential victims. Many participants understand that centering the victim is important. At the same time, faculty members find it more accessible and less intimidating to take a compassionate stance toward the source of microaggression, especially if the source holds some power over the participants. The power may be positional when the source is a senior colleague, or relational when the source is a long-time patient. During the simulated interactions, participants gained experience in navigating the tension between reaching out to the source with curiosity and compassion and holding them accountable for causing harm. The discussion about “calling-in” or “calling-out” was rich and helpful for the participants.

“Um, I think you know in the tools provided you know, definitely it's seemed like it was initially a fairly gentle approach. And it tends to be kind of open-minded and questioning and trying to make a safe space to encourage kind of change in perspectives. I really liked that approach.” (P5).

“I think mostly the message from the mediators about how being judgemental …is just not helpful…not to make assumptions that everybody is maybe on the same page as you. And that there is variability on people's opinions and that is why … the inquiry piece comes up.” (P6).

“So, I am very cautious about pouncing… I have been pounced on. I have been the recipient of that. And I am like is there any other way you could've said that. I am actually not an evil person. (Laughing). Could you say that differently because you've really deflated me. I feel completely shamed by. (Laughing). So, I feel shamed, you know.” (P9).

“But I was wondering if there was sort of a compassionate accountability that you could connect, treating a person that in a way that is kind of like compassionate. But also holds their feet to the fire with what they are saying.”(facilitator, focus group meeting transcript).

## Discussion

While undergraduate and postgraduate health sciences curricula are integrating EDI-IR principles, many teaching faculty members have not received this training. This mismatch may cause disillusionment and cynicism for the learners, and discomfort or demoralization for the faculty. More importantly, it can continue to perpetuate harm to learners and patients with marginalized identities.

Our grassroots solution to this problem is the CBS workshop. What we uncovered during our program evaluation project reveals that interrupting microaggressions in the clinical teaching environment is a complex task that involves multiple competencies. Faculty members must be sensitized to the prevalence and the harm of microaggressions. They must be skilled to act swiftly in the moment. They must be able to navigate high pressure and heightened emotions while doing so. As one of our facilitators mentioned in the focus group meeting, it is not something a faculty member could do “on autopilot.” Despite the challenges, the participants’ responses and iterative performances during the workshop give us much to be hopeful about.

By the end of 2024, our team has succeeded in delivering fifteen live workshops. We engaged 275 faculty and staff members. We have facilitated many affirming and challenging conversations. We have produced a collection of cases covering five major topics in EDI-IR. By inviting diverse expertise, the core CBS team grew in experience and skills in our facilitation. We also made new connections within our institution. We received multiple requests for future offerings. We relish the incidences when participants reported how they successfully managed microaggressions after attending the workshop. We also have each achieved our own goals in contributing to an equitable and inclusive workplace.

However, we are aware that what we have accomplished is only a small and imperfect first step towards inclusive excellence. In follow-up conversations with workshop participants, we hear requests to practice how to empower themselves as victims, or how to manage incidents where they find themselves as the source of microaggressions. We are also asked by participants who hold leadership roles to consider creating cases for leadership training beyond the day-to-day environment. These are important objectives that would require ongoing institutional investment to address in full.

It is also important to recognize that beyond grassroots projects like ours, institution leadership is concurrently doing the critically important work of applying an anti-oppression lens to existing policies and procedures and disrupting oppressive infrastructure from within. At McMaster, new Associate Deans in Equity and Inclusion was recruited in 2023 and has made headways in addressing structural changes at the Faculty of Health Sciences while collaborating with the Associate Dean in Indigenous Health [23]. We are also mindful that, as self-regulated and privileged professionals, we must grapple with the challenge of expanding the reach of these initiatives to those who do not prioritize learning in this area. Although we may have impacted hundreds of faculty members who attended, we have thousands of faculty members at our institution alone who have not. Beyond the usual constraints of time and resources, we estimate that some faculty members do not recognize their learning needs and would not voluntarily invest in learning in this topic. It is also possible that some faculty members find the topic uncomfortable to engage with, and as a result, avoid it altogether. With the voluntary and self-directed nature of faculty development, we may never reach these faculty members. It is the responsibility of faculty developers, with institutional support, to design educational activities that are accessible and engaging. We also look to our institutions and academic departments for both continued support for projects like ours and strong incentives for our colleagues in participation for their teams’ performance and their own growth. When we include more diverse perspectives in our conversations, the debriefing conversations may become increasingly challenging during workshops. Facilitators for future workshops must be uniquely knowledgeable in equity and inclusivity topics, and highly skilled in navigating these interactions. It is our belief that joining in difficult conversations on this topic is an essential first step toward achieving inclusive excellence.

### Limitations

CBS workshop participation as well as research participation is voluntary. The number of study participants is small. Family medicine teachers are prominently represented among the study participants. As such, the findings are vulnerable to selection bias.

The data was generated from participants’ and team members’ reports, thus vulnerable to recall bias and social desirability bias.

## Conclusions

Our program evaluation study shows that simulation-based education can be leveraged well for training faculty members in managing microaggressions in the clinical teaching environment. Overall, the participants found the experience effective and empowering.

We recognize the limitations of our findings and the significant amount of work that lies ahead to operationalize inclusive excellence in health professions education.

## Data Availability

•The data that support the findings of this study are stored in a secure shared drive at McMaster University. It is available from the authors upon reasonable request.
